# SCC of 2304 Duplex Stainless Steel—Microstructure, Residual Stress and Surface Grinding Effects

**DOI:** 10.3390/ma10030221

**Published:** 2017-02-23

**Authors:** Nian Zhou, Ru Lin Peng, Mikael Schönning, Rachel Pettersson

**Affiliations:** 1 Department of Material Science, Dalarna University, SE-79188 Falun, and KTH, SE-10044 Stockholm, Sweden; 2 Department of Management and Engineering, Linköping University, SE-58183 Linköping, Sweden; ru.peng@liu.se; 3 Corrosion Department, Avesta Research Center–Outokumpu Stainlelss AB, SE-77422 Avesta, Sweden; mikael.schonning@outokumpu.com; 4 Jernkontoret, SE-11187 Stockholm, and KTH, SE-10044 Stockholm, Sweden; rachel.pettersson@jernkontoret.se

**Keywords:** stress corrosion cracking, duplex stainless steel 2304, microstructure, residual stress, grinding

## Abstract

The influence of surface grinding and microstructure on chloride induced stress corrosion cracking (SCC) behavior of 2304 duplex stainless steel has been investigated. Grinding operations were performed both parallel and perpendicular to the rolling direction of the material. SCC tests were conducted in boiling magnesium chloride according to ASTM G36; specimens were exposed both without external loading and with varied levels of four-point bend loading. Residual stresses were measured on selected specimens before and after exposure using the X-ray diffraction technique. In addition, in-situ surface stress measurements subjected to four-point bend loading were performed to evaluate the deviation between the actual applied loading and the calculated values according to ASTM G39. Micro-cracks, initiated by grinding induced surface tensile residual stresses, were observed for all the ground specimens but not on the as-delivered surfaces. Loading transverse to the rolling direction of the material increased the susceptibility to chloride induced SCC. Grinding induced tensile residual stresses and micro-notches in the as-ground surface topography were also detrimental.

## 1. Introduction

Duplex stainless steels (DSS), consisting of approximately equal amount of austenite and ferrite, often combine the beneficial features of austenitic and ferritic stainless steels and today are attracting more interest for service in aggressive environments. They provide good mechanical properties and excellent resistance to corrosion. Generally, duplex stainless steels have a yield strength up to twice that of austenitic stainless steels and also exhibit higher resistance to stress corrosion cracking (SCC) in chloride environments than their austenitic counterparts [1].

SCC of stainless steels can cause failure due to the simultaneous interaction of three critical factors—a susceptible alloy, a corrosive environment and a threshold tensile stress [2]. For duplex stainless steels, the higher resistance to SCC is related to their microstructure; both the crystal structure and the chemical composition of the constituent phases are different. One of the simplest and straightforward explanation is that the two phases have completely different slip systems so it is more difficult for a crack in duplex materials to cross the phase boundary than the grain boundary in single phase materials [3]. Shimodaira et al. [4] argued that crack propagation in the austenite was hindered by the ferrite at low applied stress, while SCC propagated through both phases at a high applied stress. The concept of an electrochemical interaction between the two phases of duplex stainless steels has also been suggested [3]; investigations indicated that the austenite is the more noble phase and therefore cathodically protected by the ferritic phase [5]. Several works have confirmed this with observations of selective dissolution of the ferrite phase [6,7,8,9,10]. Another factor contributing to the high stress corrosion resistance is believed the internal stress state in duplex stainless steels. The two phases have different thermal expansion coefficients, thus residual micro-stresses are always present in duplex stainless steels [11], often compressive residual stresses in the ferritic phase, which is the matrix phase [12,13].

A number of loading methods can be used to evaluate susceptibility to stress corrosion cracking. Four-point bend loading, described in both ASTM G39 [14] and ISO 7539 [15], is widely used for strip materials in the elastic deformation regime. However, the actual surface stresses in the specimens may deviate appreciably from the calculated values using the formula according to the standards. It has been demonstrated that the actual surface stresses were largely affected by the presence of residual stresses or the occurrence of local plasticity when loading the 304L austenitic stainless steel [16]. When fabricating stainless steels, grinding is a widely used surface finishing process. Grinding induces plastic deformation and generates thermal energy, both of which lead to changes in the surface integrity of the material and may affect the service performance, including the risk for stress corrosion cracking. An increase in the corrosion rate due to surface strains associated with surface working has been reported for lean DSS bars [17]. The effectiveness of retarding stress corrosion cracking of 2205 duplex stainless steel by shot peening with different shot sizes has been demonstrated by Al-Obaid [18], who attributed this to the generation of compressive residual stresses in the surface. A high level of cold work has been reported to decrease the stress corrosion cracking resistance of UNS S32550 duplex stainless steel [19]. Dealloying in the nanocrystalline layer by surface grinding and subsequent cracking of the dealloyed layer have been observed in 22Cr and 25Cr duplex stainless steels after testing under evaporative seawater conditions [20].

Although numerous works have addressed stress corrosion cracking susceptibility of duplex stainless steels, little research has been performed to investigate the role of surface integrity in crack initiation and propagation. Hence the aim of this work is to study the chloride induced stress corrosion behavior of as-delivered and surface ground 2304 duplex stainless steel. Specimens have been exposed without external loading and under four-point bend loading. Both the grinding operations and the four-point bending have been performed in two directions, parallel and perpendicular to the rolling direction of the material. 

## 2. Experimental

### 2.1. Material

**Figure 1:**
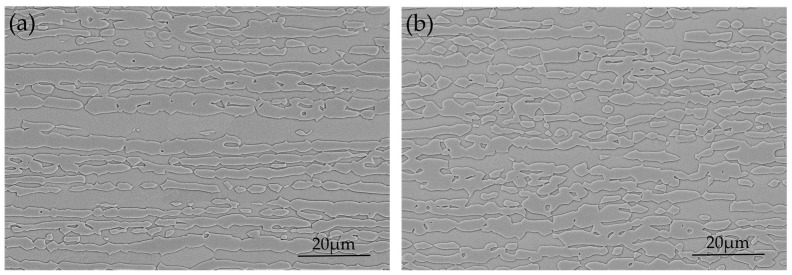
Cross-section microstructure of DSS 2304 as received showing austenitic islands in the ferritic matrix: (**a**) parallel to rolling direction; and (**b**) perpendicular to rolling direction.

The material investigated in this study was grade 2304 duplex stainless steel supplied as test coupons with 400 mm × 150 mm × 2 mm in dimensions. The as received material had a 2B surface finish and had been solution annealed (1100 °C, forced air and water quenched), pickled and roll leveled. Figure 1 shows the cross-section microstructures of this material both parallel and perpendicular to the rolling direction. The volume fraction of austenite was evaluated as 46.8% and that of ferrite as 53.2% using the electron backscatter diffraction (EBSD) technique. The chemical composition and macroscopic mechanical properties measured perpendicular to the rolling direction at room temperature are given in Table 1 and Table 2, respectively.

### 2.2. Grinding Operation

The grinding operations were conducted on a Chevalier FSG-2A618 grinding machine with the grinding belt mounted on a grinding wheel; grinding belts (50 mm in width, 473 mm in length) with conventional aluminum oxide grit were used. The detailed grinding set-up is described in [13]. A fixed grinding speed v_s_ = 23 m/s, a fixed feed rate v_w_ = 8 m/min and a fixed motor power of 600 W were used. Grinding was first performed for 2.5 min with 60# grit size abrasive to remove the as-delivered material surface, and then followed by another 2.5 min grinding using a new 60# grit size abrasive to get the final surface finish. No grinding lubricant was used during the whole process. The grinding operations were performed both parallel to and transverse to the rolling direction of the material.

### 2.3. Corrosion Tests

The susceptibility to chloride induced stress corrosion cracking was tested both without external loading and under four-point bend loading conditions. Boiling magnesium chloride solution according to ASTM G36 [21] was used as the test environment and the temperature carefully controlled to 155 °C ± 1 °C. Prior to testing, all the edges of specimens were ground down by 1000# grinding paper to avoid rough and sharp edges; then the specimens were allowed to passivate in air for at least 24 h before exposure.

**Figure 2:**
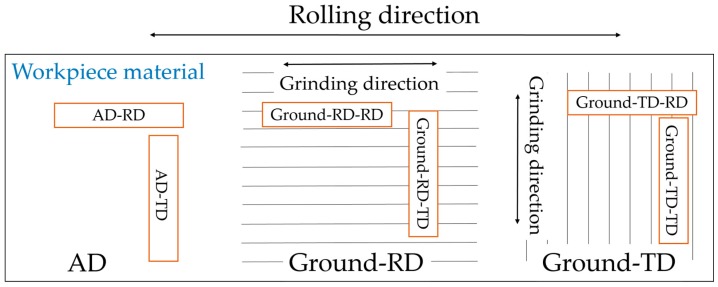
Schematic illustration of the different specimens tested. The orange rectangles show the geometry of the four-point bending specimens with loading applied along the longitudinal direction. AD is as-delivered material, Ground-RD denotes material ground parallel to the rolling direction while Ground-TD has been ground in the transverse direction. The second part of the specimen designation denotes the loading direction (RD or TD).

As illustrated in Figure 2, three groups of specimens were tested. The as-delivered material, denoted AD; ground specimens with grinding performed parallel to the rolling direction, denoted Ground-RD and ground transverse to the rolling direction, denoted Ground-TD. The first set of tests was without applying any external loading to evaluate the effect of residual stresses. Duplicate specimens were tested for each condition. They were cut as 30 mm × 30 mm square samples and exposed for 20 h.

In the second series of tests, four-point bend loading was applied to specimens according to ASTM G39 [14]. Specimens were cut with dimensions 65 mm × 10 mm. Totally, six types of specimens were tested, as shown in Figure 2. The as-delivered material cut with the long axis parallel or perpendicular to the rolling direction, are denoted AD-RD or AD-TD. The Ground-RD-RD and Ground-RD-TD are specimens cut along and transverse to the rolling/grinding directions of the Ground-RD material. Similarly, specimens cut parallel and transverse to the original rolling direction (which is perpendicular to the grinding direction) of the Ground-TD specimen, are denoted Ground-TD-RD and Ground-TD-TD respectively. For all the specimens, the loading direction was along the long axis of the specimen. Before exposure, each specimen was kept for one hour in air to allow possible stress relaxation after the application of loading. In this case, the exposure time was 22 h.

### 2.4. Material Characterization

The specimens were checked for the presence of macro-cracks after exposure using a stereo microscope (Nikon SMZ-2T with ColorView Soft camera and Cell^A^ ColorView soft image software) at ×10 to ×63 magnification. SEM (Scanning electron microscopy, FEG-SEM Zeiss Ultra 55) was used to investigate the surface topography. Cross-sectional investigation of selected specimens after exposure were performed from both longitudinal (LD) and transversal (TD) directions. In addition, some fracture surfaces were examined.

The in-depth residual stress profiles parallel (σ_‖_) and perpendicular (σ_⊥_) to the rolling directions were determined by X-ray diffraction for both AD and Ground-RD specimens. Cr-$K_{\alpha }$ radiation was used to determine the elastic strains in the {220} lattice planes of the austenitic phase (diffraction peak at 2θ ~ 128°) and the {211} lattice planes of the ferritic phase (diffraction peak at 2θ ~ 154°), respectively. Typical information depth in the analysis was estimated to be about 5 µm [22]. Residual stresses were calculated based on the sin^2^ψ method under the assumption of plane stress condition [22]. The X-ray elastic constants, S_1_ and 1/2S_2_, used for the stress calculations are 1.2 × 10^−6^ MPa^−1^ and 6 × 10^−6^ MPa^−1^ for austenite and 1.25 × 10^−6^ MPa^−1^ and 5.58 × 10^−6^ MPa^−1^ for ferrite. Controlled electrolytic polishing was used to remove material in order to measure the in-depth profiles; no correction was made for possible stress relaxation due to material removal. Surface stresses of both Ground-RD and Ground-TD specimens were also measured parallel to the grinding direction before and after exposure without any external loading using the same method. In addition, in-situ surface stress measurements were made on selected specimens subjected to loading in the same four-point bending fixtures which were used for the stress corrosion cracking tests. The applied loading was calculated according to the following equation from ASTM G39 [14]:

$$\sigma =12Ety/(3H^{2}-4A^{2})$$

where

σ = maximum tensile stress;

*E* = modulus of elasticity;

*t* = thickness of specimen;

*y* = maximum deflection (between outer supports);

*H* = distance between outer supports; and

*A* = distance between inner and outer supports.

The applied loading was increased in steps to 10, 300, 500, 700 and 900 MPa. It should be noted that the two higher values are above the measure yield stress of the as-delivered material. They are thus outside the range for which the calculation formula [14] is valid and the actual stresses may be expected to deviate appreciably from the calculated values due to yielding. After each loading was applied, the specimen was kept one hour under the load for stress relaxation, and then the actual surface stresses parallel to the loading direction were measured by X-ray diffraction.

## 3. Results

### 3.1. Pre-Corrosion Characterization

#### 3.1.1. Surface Topography and Surface Roughness

**Figure 3:**
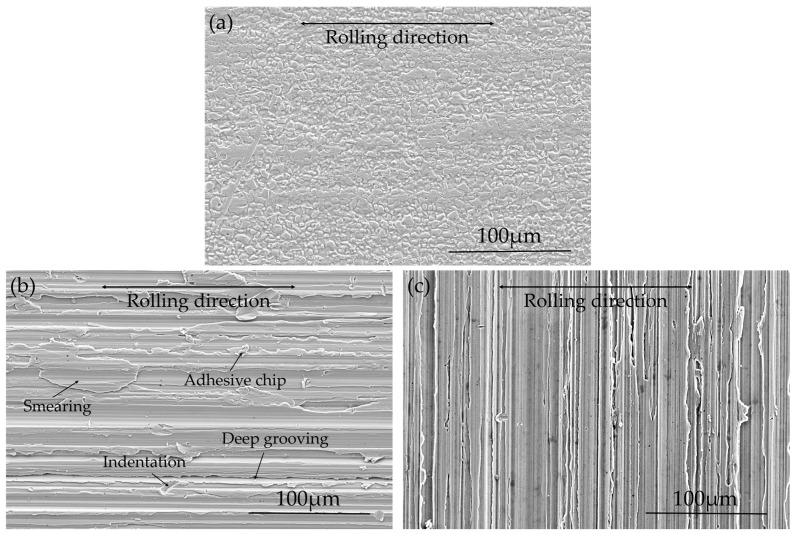
Surface topography of” (**a**) AD specimen; (**b**) Ground-RD specimen; and (**c**) Ground-TD specimen.

Surface topography of AD, Ground-RD and Ground-TD specimens before exposure is shown in Figure 3. Figure 3a, which is the as-delivered 2B surface, shows that the pickling process during production slightly etched the grain boundaries. For the ground specimens, as shown in Figure 3b,c, surface defects including deep grooving, smearing, adhesive chips and indentations were observed after both parallel and transversal grinding. The generation of such surface defects have been described in detail in our previous work [13]. Measured surface roughness, in terms of both R_a_ and R_z_ values, are listed in Table 3. The surface roughness was measured using a 3D optical topometer (Wyko NT9100); the results are the average of five measured areas (1.3 mm × 0.95 mm) and the standard deviations are calculated from the five measurements for each sample. As shown from the table, surface roughness increased largely by both parallel and transverse grinding.

#### 3.1.2. In-Depth Residual Stresses

**Figure 4:**
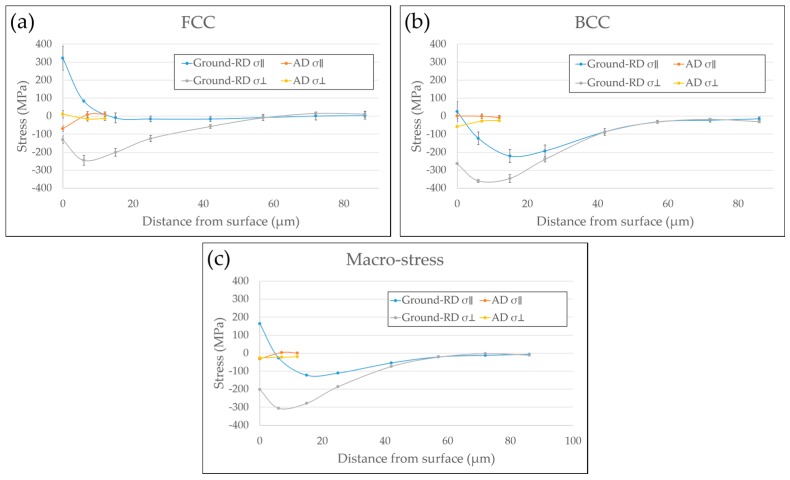
In-depth residual stresses of AD and Ground-RD specimens: (**a**) residual stresses in austenitic phase; (**b**) residual stress in ferritic phase; and (**c**) macro-stress. Positive values denote tensile stresses and negative compressive stresses.

The in-depth residual stress profiles parallel (σ_‖_) and perpendicular (σ_⊥_) to the rolling directions of both AD and Ground-RD specimens were presented in a previous paper [13], in which the Dölle-Hauk method was used for calculating the full stress tensor (3D residual stress). In order to compare with the 2D stress analysis under in situ bending done in the current work, the XRD results from [13] were reanalyzed using the 2D method and the obtained residual stress profiles are presented in Figure 4, It should be noted that the resulting differences between the 3D and 2D analyses are within the uncertainty range of the stress values. The results are presented as phase stresses and macro-stress. Phase stresses are residual stresses measured in the austenitic phase (FCC), σ^γ^, and ferritic phase (BCC), σ^α^, respectively. Macro-stresses, which are homogeneous residual stresses on a macroscopic scale [22], were calculated according to the equation: σ^M^ = V^γ^σ^γ^ + (1 − V^γ^)σ^α^, where V^γ^ is the volume fraction of the austenitic phase in the material. As shown in Figure 4, for both phase stresses and macro-stresses, low levels of residual stresses from surface layer to subsurface region were observed in the AD specimen in both directions. However, the grinding operation generated tensile macro σ_‖_ but compressive macro σ_⊥_ in the surface layer, as seen in Figure 4c. The macro tensile σ_‖_ were highest (above 150 MPa) in the ground surface, and dropped rapidly to compression within around 5 µm in depth. The compressive macro σ_⊥_ showed a relatively low value in the surface, but increased rapidly and reached a peak value of over 300 MPa in the subsurface region. The phase stresses in Figure 4a,b revealed different trends of grinding induced residual stresses between the austenitic phase and the ferritic phase. In the austenitic phase, tensile σ_‖_ but compressive σ_⊥_ were observed; the tensile σ_‖_ were highest in the surface, over 300 MPa, and decreased gradually from surface to subsurface. In the ferritic phase, except for the small tensile surface σ_‖_, grinding induced compressive residual stresses in both directions with a peak value in the subsurface; σ_⊥_ were more compressive than σ_‖_.

For the Ground-TD specimen, similar trend of residual stress states were measured in the ground surface, however, the depth profiles were not obtained.

#### 3.1.3. In-Situ Measured Surface Stresses under Four-Point Bend Loading

**Figure 5:**
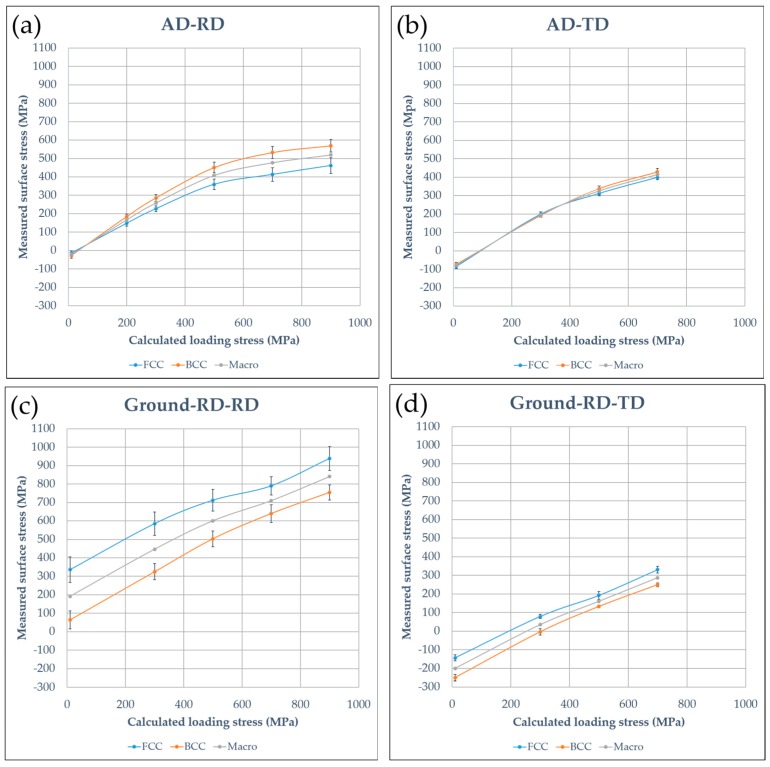

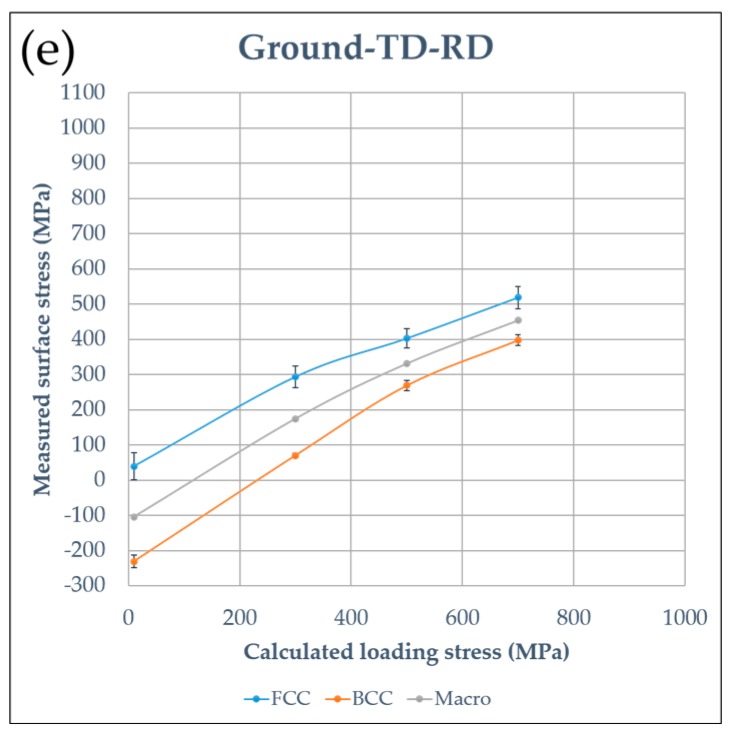
In-situ measured surface stresses along loading direction under four-point bend loading of: (**a**) AD-RD specimen; (**b**) AD-TD specimen; (**c**) Ground-RD-RD specimen; (**d**) Ground-RD-TD specimen; and (**e**) Ground-TD-RD specimen. Positive values denote tensile stresses and negative compressive stresses.

In-situ measured surface stresses of AD-RD, AD-TD, Ground-RD-RD, Ground RD-TD and Ground-TD-RD specimens subjected to four-point bend loading along the loading direction are presented in Figure 5. Stresses before the loading agree well with Figure 4, although there are small differences for the AD specimen which may be related to specimen position or geometry. As shown in Figure 5a,b, different trends have been observed when loading applied parallel or perpendicular to the rolling direction of the as-delivered material. For the AD-RD specimen (Figure 5a) the actual surface stresses in the ferritic phase were close to the calculated loading in the elastic regime; above the yield stress, the slope of the curve decreased significantly. Lower surface stresses were measured in the austenitic phase over the whole loading range, and the difference increased with increasing load. However, when applying the loading transverse to the rolling direction (AD-TD specimen, Figure 5b), similar values of surface stresses were measured in both phases before yielding; but the actual stresses were lower than the calculated values. Above the proof stress, the slope of the curve dropped, and the actual stresses showed some difference between the two phases, with a higher value in the ferritic phase. In the case of ground specimens, (Figure 5a–c), the actual measured surface stresses were lower than the grinding induced surface stresses plus the calculated loading values in the elastic regime; different level of stresses were measured in the two phases, respectively, and the slope of the ferritic phase was a little higher than that of the austenitic phase. Above the yield stress, a decrease of the slope of the austenitic curve was observed to correspond to the increased slope for the ferritic curve. 

### 3.2. Corrosion Behavior without External Loading

#### 3.2.1. Surface Morphology after Exposure

**Figure 6:**
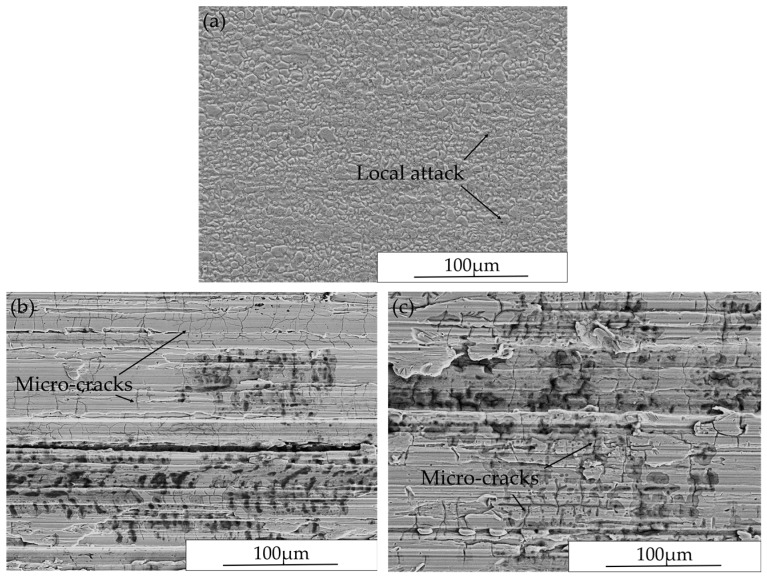
Surface morphology after exposure without external loading of: (**a**) AD specimen; (**b**) Ground-RD specimen; and (**c**) Ground-TD specimen.

Figure 6 contains SEM images showing the surface morphology after exposure. Macro-cracks were not found on any specimen after exposure regardless of surface conditions. In this study, macro-cracks are defined as those that can be observed by stereo microscopy with ×63 as highest magnification. However, extensive branched micro-cracks were present in all ground specimens (Figure 6b,c) even without applying any external loading. For both Ground-RD and Ground-TD specimens, the micro-cracks were primarily oriented perpendicular to the grinding marks. This correlates to the high level tensile macroscopic residual stresses measured parallel to the grinding direction in the surface layer. In addition, extensive pitting was associated with these micro-cracks. In the case of AD specimen (Figure 6a), no micro cracking occurred, although a few small areas of slight corrosion were present.

#### 3.2.2. Cross-Section Investigation after Exposure

**Figure 7:**
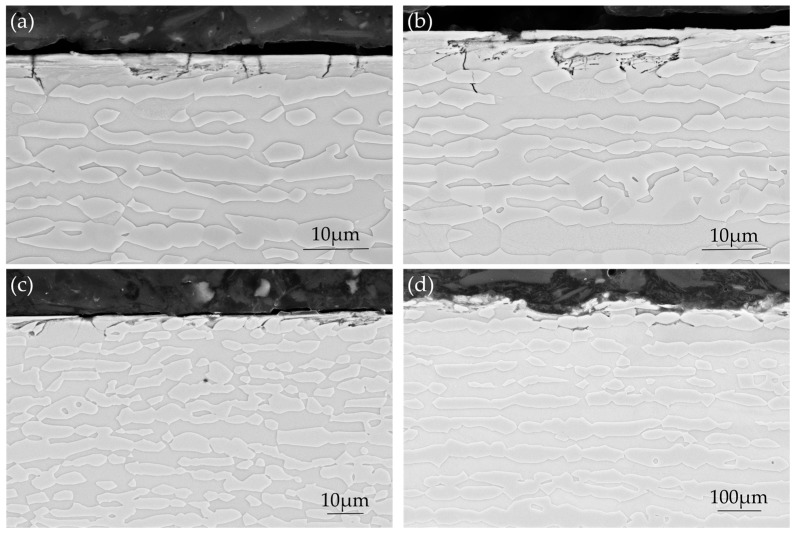
Cross-section microstructures after exposure without external loading: (**a**,**b**) Ground-RD specimen sectioned parallel to rolling direction; (**c**) Ground-TD specimen sectioned perpendicular to rolling direction; and (**d**) Ground-TD specimen sectioned parallel to rolling direction.

Typical SEM images of cross-section microstructures of ground specimens after exposure without external loading are presented in Figure 7. As illustrated in Figure 7a,c, micro-cracks appeared mainly perpendicular to the grinding marks for both Ground-RD and Ground-TD specimens, which agrees with the surface morphology investigation. Micro-cracks were observed to have initiated and propagated in both phases. Grinding operations generated a heavily deformed surface layer with a thickness of a few micrometers, which has been described in detail in previous work [13]. The cracks initiated from the ground surface and most of them were within this highly deformed surface layer. In addition, cracks also tended to be arrested at phase boundaries. Some selective dissolution of the ferritic phase started from these micro-cracks and extended in both directions, as shown in Figure 7b,d.

#### 3.2.3. Stress Release after Exposure

**Figure 8:**
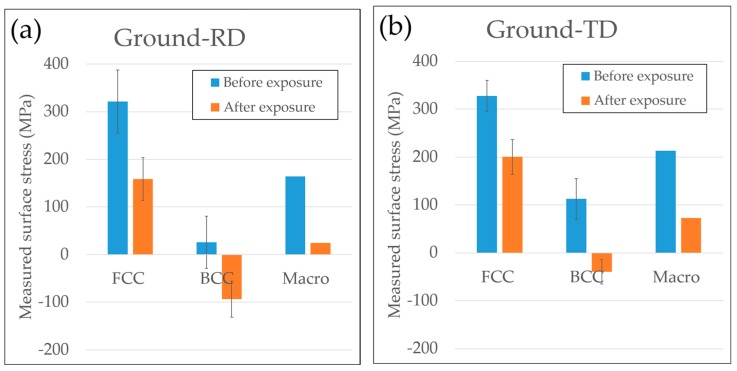
Surface stresses of ground specimens parallel to grinding direction before and after exposure without external loading: (**a**) Ground-RD specimen; and (**b**) Ground-TD specimen

Measured surface stresses along the grinding direction of both Ground-RD and Ground-TD specimens before and after exposure without external loading are compared in Figure 8. Both parallel and transverse grinding induced tensile surface residual stresses along the grinding direction, and these stresses were higher in the austenitic phase than the ferritic phase. After 20 h exposure without applying any external loading, surface tensile stresses reduced significantly for both specimens. This can be explained by micro-cracks releasing the surface tensile stresses. 

### 3.3. Corrosion Behavior with Four-Point Bend Loading

#### 3.3.1. Macro-Crack Examination

Macro-cracks were observed to develop in some specimens after exposure under four-point bend loading, Table 4 summarizes the occurrence of micro- and macro-cracks after corrosion testing with different applied loads. When the loading was applied parallel to the rolling direction of the material, the results showed that one out of three AD-RD specimens cracked through the thickness after 22 h exposure in boiling MgCl_2_ at 900 MPa loading. Both parallel grinding (Ground-RD-RD) and transverse grinding (Ground-TD-RD) lowered the threshold stress for SCC to 700 MPa. In the case of an applied load perpendicular to the rolling direction, one out of three AD-TD specimens cracked when the applied load was only 500 MPa; increasing the load to 700 MPa gave macro-cracks on all the exposed specimens. Parallel grinding (Ground-TD-TD) increased the susceptibility to stress corrosion cracking while transverse grinding (Ground-RD-TD) reduced the susceptibility; after exposure under 500 MPa loading, two out of three Ground-TD-TD specimens cracked while no macroscopic cracking was observed on the Ground-RD-TD specimens. In the Discussion, the concept of a threshold stress for cracking is used; this is taken as being the lowest macroscopic stress in the loading direction at which macro cracking is observed, i.e., for AD-RD it is nominally 900 MPa and actually 520 MPa.

#### 3.3.2. Surface Morphology after Exposure

**Figure 9:**
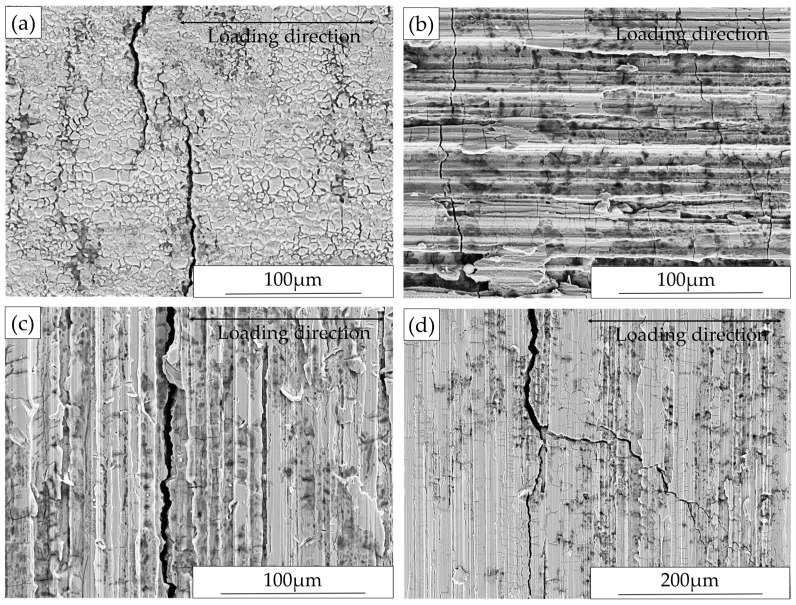
Surface morphology after exposure under four-point bend loading: (**a**) AD-RD specimen at 900 MPa; (**b**) Ground-RD-RD specimen at 700 MPa; (**c**) Ground-RD-TD at 700 MPa; and (**d**) Ground-TD-RD specimen at 700 MPa.

Surface investigation after exposure frequently showed multiple cracks with one major crack nearly through the specimen thickness. SEM micrographs with detailed characterization of selected specimen surfaces are given in Figure 9. The macro-cracks were wide with multiple branching; they were primarily perpendicular to the loading direction and tended to run parallel to each other. Similar to specimens exposed without external loading, extensive branched micro-cracking mainly oriented perpendicular to the grinding marks were found on all the exposed ground surfaces regardless of the grinding direction, the loading direction, the loading level or the occurrence of macro-cracking. However, this kind of attack was not observed on the as-delivered material surfaces after exposure. Varied degrees of pitting were observed for all specimens; cracks were sometimes seen associated with pits. For the ground specimens with loading applied parallel to the grinding marks, as shown in Figure 9b, pitting showed some tendency to grow from the micro-cracks and connected together perpendicular to the loading direction. For specimens loaded perpendicular to the grinding direction (Figure 9c,d), pits were observed at both micro-cracks and deep grooves, and could grow together along the grooves.

#### 3.3.3. Cross-Section Investigation after Exposure

**Figure 10:**
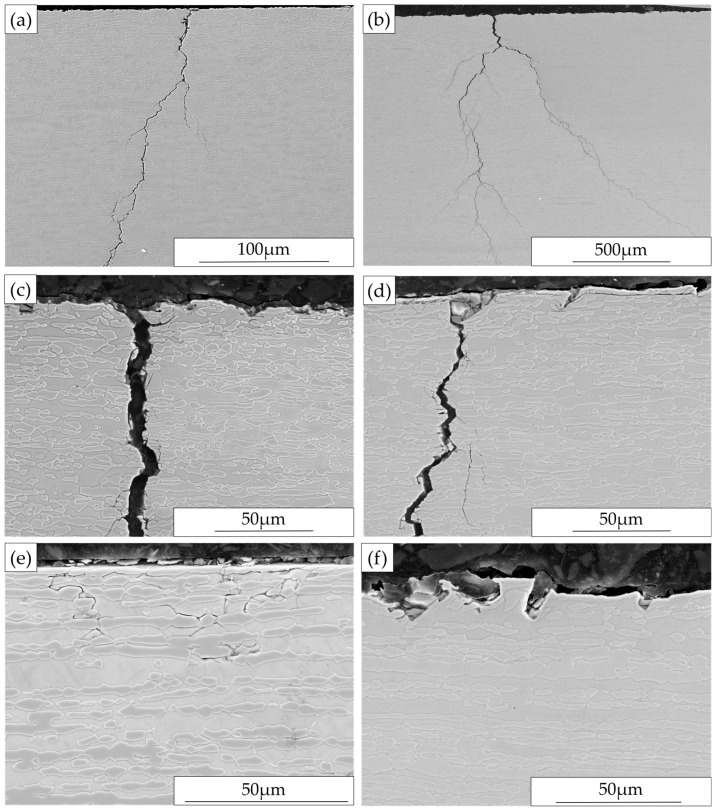
Cross-section microstructures showing stress corrosion cracking after exposure under four-point bend loading: (**a**) AD-TD specimen, 700 MPa, sectioned parallel to loading direction; (**b**,**c**) Ground-RD-TD specimen, 700 MPa, sectioned parallel to loading direction; (**d**) Ground-TD-TD specimen, 500 MPa, sectioned parallel to loading direction; (**e**) Ground-RD-TD specimen, 700 MPa, sectioned perpendicular to loading direction; and (**f**) Ground-RD-RD specimen, 900 MPa, sectioned parallel to loading direction.

Cross-sections parallel and perpendicular to the loading direction were examined for some specimens, and selected SEM images are presented in Figure 10 as examples. As shown in Figure 10a,b, the macro-cracks appeared highly branched. In the sections parallel to loading direction (Figure 10c,d), the path of micro-cracks was mainly transgranular and they were observed to propagate in both the austenitic and the ferritic phases. However, in the section perpendicular to loading direction, as shown in Figure 10e, cracks with much smaller penetration depth were observed; they propagated mainly in ferrite, which is the continuous phase and followed the austenitic-ferritic phase boundaries. Angular pits or corroded crack openings were observed particularly at higher applied loads, and corrosion products or residual grinding products were sometime present in these areas, as illustrated in Figure 10d,f. The size and number of these corroded areas increased with increasing applied load and they were more frequent in specimens with grinding perpendicular to the loading direction. Around the corroded areas, material was observed to be heavily deformed. This kind of attack was not observed in specimens with as-delivered surface condition; only selective dissolution of the ferritic phase in a very thin layer from the surface was found.

#### 3.3.4. Fracture Surface Investigation

**Figure 11:**
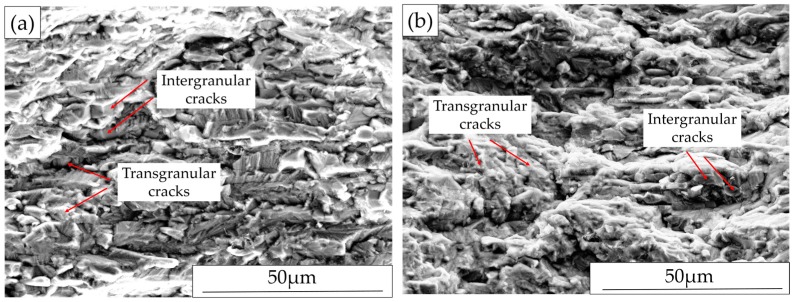
Fracture surface after exposure: (**a**) AD-TD specimen under 700 MPa loading; and (**b**) Ground-RD-RD specimen under 900 MPa loading.

Typical fracture surfaces are presented in Figure 11. The SEM images showed fracture appeared predominantly transgranular, although some local intergranular cracking were observed. In addition, crack branching can be seen from both specimens. The results agree with the cross-sectional investigations.

## 4. Discussion

### 4.1. Corrosion Behavior without External Loading

In the absence of any external loading, extensive stress corrosion micro-cracks were observed on the ground surfaces, while the as-delivered surfaces showed no such attack. Similar cracking has also been observed on both ground [16] and milled [23] austenitic stainless steel surfaces. In the current work, measured residual stresses for the as-delivered material were close to zero; however, the grinding operations generated large anisotropic residual stresses in the surface and sub-surface layers of the material. Different levels of residual stresses were observed in the austenitic and ferritic phases. The generation and distribution of residual stresses in this ground duplex material have been discussed in detail in previous work [13]. It has been proposed that surface tensile residual stresses from the machining processes [16,23] can promote stress corrosion cracking, while compressive residual stresses can delay the crack initiation or slow down the crack growth from surface [16,24].

In the present work, tensile residual stresses over 300 MPa were measured in the austenitic phase along the grinding direction in the surface layer. Although much lower levels were measured in the ferritic phase, the overall macroscopic tensile residual stresses were around 200 MPa for both Ground-RD and Ground-TD specimens. Both the surface morphology and cross-section investigations after exposure showed micro-cracks appeared mainly perpendicular, i.e., perpendicular to the maximum tensile stress induced by grinding. Thus, the tensile residual stresses in the ground surface renders the duplex stainless steel susceptible to microcracking in presence of chloride even without applying any external loading. The micro-cracks initiated and propagated in both phases but the penetration depth was small and the majority of cracks were within the highly deformed ground surface layer. This correlates to the position at which the macroscopic residual stresses shift from tension to compression. The formation of micro-cracks caused significant release of the surface tensile residual stresses. Selective dissolution of the ferritic phase was observed and tended to start from the micro-cracks initiated in the ferrite and grow in both directions. This correlates to reported observations that corrosion potential of the ferritic phase is lower than that of the austenitic phase in chloride containing media, which led to a selective attack in the ferrite [10].

### 4.2. Measured Surface Stresses during In-Situ Four-Point Bend Loading

The in-situ measurements on surface stresses under four-point bend loading demonstrated that the actual surface stresses of specimens can deviate very significantly from the values calculated according to ASTM G39 [14]. The actual surface stresses were affected by the surface preparation and were different for the austenite and ferrite phases. The two phases have different elastic and plastic properties, thus their interaction can give rise to complex flow behavior during deformation [25]. The banded microstructure and anisotropic material properties from hot or cold rolling also contribute to a complex partitioning between the two phases during loading [26].

For the as-delivered material loaded parallel to the rolling direction (specimen AD-RD), i.e., parallel to the direction in which the ferrite and austenite phases are elongated, a simple model is that the two phases experience the same strain (Voigt model) [27] in the surface layer. However the different Young’s Modulus means that different stress levels develop in the two phases with higher actual surface tensile stresses in the ferritic phase and lower in the austenitic phase over the whole loading range, Figure 5a. For the AD-TD specimen the banding of the microstructure is perpendicular to the loading direction and it is more appropriate to assume the Reuss model (isostress) [27]. This means that similar stresses are attained in both phases until the plastic deformation regime is reached. This agrees with the measured behavior seen in Figure 5b. For both specimens, from the macroscopic point of view, the actual loading corresponded well with the calculated values from ASTM G39 in the elastic regime, although the measured actual surface macro-stresses were lower for the AD-TD than the AD-RD specimen, which is due to the initially higher compressive residual surface stress perpendicular to the rolling direction. Above a certain loading, local yielding occurs in the surface or subsurface, in one or both phases, depending on both the local strength and residual stresses. The relaxation of local residual stresses also results in redistribution of both the residual stresses and applied stresses and thus can be expected to affect the surface phase stresses/macrostress evolution [28]. With increasing loading, the extent of plastic deformation and stress redistribution will increase so the calculated stresses from four-point bending will not be attained. For this reason, the discussion in the following section is primarily in terms of the actual measured stresses. As described in ISO 16540 and NACE TM316-2016, calibration using the uniaxial stress-strain data or testing in the overstrained condition can be performed [29,30] but was outside the scope of the present study.

Different trends of in-situ surface stress measurements were observed for the ground specimens. The surface layer was highly deformed so the actual measured surface stresses depend on the interaction between the applied and the residual stresses as well as the strength gradient under the surface [28]. Grinding operations generated predominantly tensile stresses parallel to the grinding direction and perpendicular compressive stresses, thus the actual measured surface stresses appreciably depending on whether the load was applied parallel or perpendicular to the grinding directions. For all the ground specimens, the actual stresses were lower than the sum of the grinding induced surface stresses and the calculated loading values, which is probably due to the grinding induced compressive residual stresses in the subsurface. Grinding induced residual stresses were also different in the austenitic and ferritic phases, leading to the different levels of actual surface stresses in the two phases after loading. The changes of the slopes of the austenitic and ferritic curves also indicated that stress redistribution occurred between the two phases after yielding. 

### 4.3. Corrosion Behavior with Four-Point Bend Loading

The macro-cracks were generally perpendicular to the loading stresses, and their path was mainly transgranular through both the austenite and the ferrite, although some local areas of intergranular cracking were observed. Along the loading direction, cracks were observed to propagate mainly in the continuous ferrite phase but the penetration depth was small (Figure 10e). This would appear consistent with the concept of suppression of cracking in the austenitic phase at low applied stress [5]. For all types of specimens, as shown in Table 4, the SCC susceptibility increased with increasing four-point bend loading.

Varying degrees of corrosion attack were observed on all the exposed specimens. For the as-delivered surface, selective dissolution of ferrite was observed and small pits were present. Due to the combination of stress concentration and a more aggressive environment in the pits [2], pits can act as precursors to cracking [16,31] alternatively subsequent corrosion may widen a crack opening into a more pit-like geometry. In the current work, macro-cracks were observed to be associated with pits, although there were pits without associated cracks, indicating that the pits formed before macroscopic cracks and acted as precursors to macro-cracking.

In the case of ground specimens, extensive micro-cracks appeared on the exposed surfaces and could be both with and without visible corrosion. This indicated the grinding induced tensile residual stress was the main driving force for the formation of micro-cracks and pitting/selective corrosion tended to grow from these micro-cracks. In addition, for the specimens with loading applied perpendicular to the grinding marks, corrosion tended to develop from these grooves/micro-notches. This corrosion behavior was very different from the as-delivered surfaces, probably due to the highly deformed ground surface layer with damage and grain fragmentation [13]. Macro-cracking was only observed on specimens under four-point bend loading, while not on specimens without external loading, indicating a sustainable tensile stress was required for the initiation and propagation of macro-cracks.

**Figure 12:**
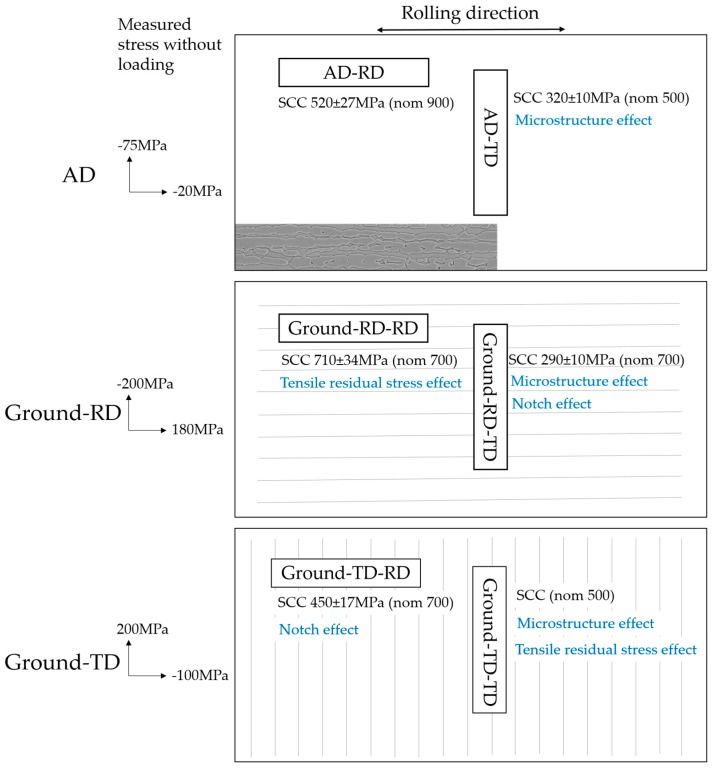
Summary of measured residual stresses and threshold stresses for macrocracking, illustrating the major factors affecting the SCC susceptibility. Positive values denote tensile stresses and negative compressive stresses.

Surface deformation, surface topography and microstructure can all influence the SCC behavior. Testing as-delivered material in two loading directions shows that the microstructure orientation has a large effect on the macro-cracking behavior. Applying the loading perpendicular to the rolling direction (AD-TD specimen) significantly increased its susceptibility to SCC compared with parallel loading (AD-RD specimen). As illustrated in Figure 12, the applied loading stress for cracking was 500 MPa in the former case and 900 MPa in the latter. The corresponding actual measured threshold surface stresses were 320 and 520 MPa, respectively. This indicates that cracking occurs more readily if there is a possibility for cracks to follow phase boundaries or remain within a single phase, as is the case for the AD-TD specimen.

Grinding increased the susceptibility to SCC; both due to grinding induced tensile residual stresses and the changes in the surface topography/micro-notches. Comparing Ground-RD-RD and Ground-TD-RD specimens with the AD-RD specimen (all loading along the rolling direction of the material), the results clearly indicated that both grinding operations increased the susceptibility to SCC. For the Ground-RD-RD specimen, the nominal threshold loading stress dropped from 900 MPa to 700 MPa, the grinding induced surface tensile residual stress is the main detrimental factor, as has been demonstrated in previous work on 304 L austenitic stainless steel [16]. However, the actual measured threshold surface stress for the Ground-RD-RD specimen was 710 MPa, compared to 520 MPa for the AD-RD specimen. This is probably due to the grinding induced compressive residual stresses under the ground surface that retarded the crack propagation.

For the Ground-TD-RD specimens, compressive residual stresses were induced in the loading direction by the grinding operation. The threshold applied loading stress at which cracking was observed was 700 MPa, but the actual measured threshold stress was only 450 MPa. The decrease in threshold stress in this case compared to the ground-RD-RD case indicates a detrimental effect of the surface micro-notches produced by grinding. Comparing the AD-TD, Ground-RD-TD and Ground-TD-TD specimens, similar results are observed although loading was applied perpendicular to the rolling direction. For the Ground-RD-TD specimen, the detrimental combination of both microstructure and micro-notches resulted in a measured threshold surface stress of only 290 MPa.

## 5. Conclusions

Investigation of the influence of surface grinding and microstructure on chloride induced stress corrosion cracking of 2304 duplex stainless steel in boiling magnesium chloride solution lead to the following conclusions:

- Grinding induced surface tensile residual stresses were found to be the main factor causing the formation of micro-cracks on ground surfaces during exposure with or without external loading. The micro-cracks arrested in the region with low or no tensile macro stresses, tended to stop at phase boundaries and caused some release of the surface stresses. Selective dissolution of ferrite tended to initiate at micro-cracks on the surface.
- The in-situ surface stress measurements on four-point bend loaded specimens demonstrated that the actual surface stresses may differ between the two phases and may deviate from the calculated values according to the formula in ASTM G39. For as-delivered material with low residual stresses, the formula gave a reasonable estimation in the elastic regime. For ground specimens, the actual surface stress depends on the interaction between the applied and the residual stresses as well as the strength gradient under the ground surface.
- Macroscopic cracking leading to final failure only appeared under an applied load. The stress corrosion macro-cracks mainly propagated transgranularly through both the austenitic and ferritic phases perpendicular to the loading direction. Along the loading direction, where the applied stress was low, they mainly propagated in the continuous ferritic phase and tended to bypass the austenite by following the austenitic-ferritic phase boundaries.
- Microstructure has a strong influence on the SCC behavior; applying loading perpendicular to the rolling direction of the material significantly increased the susceptibility to SCC. Grinding induced tensile residual stresses and micro-notches from grinding also have a detrimental effect.

## Figures and Tables

**Table 1:** Chemical composition (wt %) of DSS 2304.

C	Si	Mn	P	S	Cr	Ni	Mo	N	Cu	Ti
0.019	0.39	1.48	0.028	0.001	23.35	4.84	0.36	0.125	0.22	0.006

**Table 2:** Macroscopic mechanical properties of DSS 2304 measured perpendicular to the rolling direction at room temperature.

*R*_p0.2_ (MPa)	*R*_m_ (MPa)	Elongation (%)	Hardness (HB)
590	739	30	228

**Table 3:** Measured surface roughness of AD, Ground-RD and Ground-TD specimens.

Specimen	Ra (μm)	Rz (μm)
AD	0.316 ± 0.0038	8.09 ± 0.56
Ground-RD	1.45 ± 0.0826	15.84 ± 2.48
Ground-TD	1.41 ± 0.0911	15.03 ± 1.98

**Table 4:** Micro- and macroscopic cracking after exposure 22 h under different four-point bend loading values; the measured surface stresses from Figure 5 are included for comparison.

Specimen	Nominal Load (MPa)	Measured Surface Stress Parallel to Loading Direction (MPa)			No. of Specimens Tested	No. of Exposed Specimens with Macro-Cracks	Presence of Micro-Cracks
		FCC	BCC	Macro			
AD-RD	700	415 ± 37	530 ± 33	475 ± 25	3	0	No
	900	460 ± 43	570 ± 34	520 ± 27	3	1	No
AD-TD	300	200 ± 11	190 ± 8	195 ± 7	3	0	No
	500	310 ± 13	335 ± 14	320 ± 10	3	1	No
	700	400 ± 12	430 ± 19	415 ± 11	3	3	No
Ground-RD-RD	700	790 ± 49	640 ± 48	710 ± 34	3	1	Yes
	900	940 ± 66	755 ± 41	840 ± 38	3	3	Yes
Ground-RD-TD	500	190 ± 5	130 ± 7	160 ± 11	3	0	Yes
	700	330 ± 18	250 ± 10	290 ± 10	3	3	Yes
Ground-TD-TD	300				3	0	Yes
	500				3	2	Yes
Ground-TD-RD	500	400 ± 28	270 ± 14	330 ± 15	3	0	Yes
	700	520 ± 32	400 ± 15	450 ± 17	3	1	Yes

## References

[1] Outokumpu (2013). Handbook of Stainless Steels.

[2] Jones D. (1996). Principles and Prevention of Corrosion.

[3] Pettersson R., Johansson E. Stress corrosion resistance of duplex grades. Proceedings of the Duplex Stainless Steel World Conference.

[4] Shimodaira S., Takano M., Takizawa Y., Kamide H. (1977). Stress Corrosion Cracking and Hydrogen Embrittlement of Iron Base Alloys.

[5] Kwon H.-S., Kim H.-S. (1993). Investigation of stress corrosion susceptibility of duplex (α + γ) stainless steel in a hot chloride solution. Mater. Sci. Eng. A.

[6] Symniotis E. (1990). Galvanic Effects on the Active Dissolution of Duplex Stainless Steels. Corrosion.

[7] Tsai W.-T., Lo I.-H. (2007). Selective dissolution and corrosion fatigure behaviors of 2205 duplex stainlelss steel. Adv. Mater. Sci..

[8] Zanotto F., Grassi V., Balbo A., Monticelli C., Zucchi F. (2014). Stress corrosion cracking of LDX 2101^®^ duplex stainless steel in chloride solutions in the presence of thiosulphate. Corros. Sci..

[9] Kahram M., Asnavandi M., Koshy P., Sorrell C.C. (2015). Corrosion Investigation of Duplex Stainless Steels in Chlorinated Solutions. Steel Res. Int..

[10] Örnek C., Engelberg D. (2016). Towards understanding the effect of deformation mode on stress corrosion cracking susceptibility of grade 2205 duplex stainless steel. Mater. Sci. Eng. A.

[11] Johansson J., Oden M., Zheng X. (1999). Evolution of the residual stress state in a duplex stainless steel during loading. Acta Mater..

[12] Moverare J.J. (2001). Microstresses and Anisotropic Mechanical Behavior of Duplex Stainless Steels.

[13] Zhou N., Lin R.P., Pettersson R. (2016). Surface Integrity of 2304 Duplex Stainless Steel after Different Grinding Operations. J. Mater. Process. Technol..

[14] ASTM International (2011). Standard Practice for Preparation and Use of Bent-Beam Stress-Corrosion Test Specimens.

[15] International Organization for Standardization (1995). Corrosion of Metals and Alloys-Stress Corrosion Testing.

[16] Zhou N., Pettersson R., Peng R.L., Schönning M. (2016). Effect of Surface Grinding on Chloride Induced SCC of 304L. Mater. Sci. Eng. A.

[17] Bautista A., Alvarez S.M., Velasco F. (2015). Selective corrosion of duplex stainless steel bars in acid. Part 2: Effect of the surface strain and numerical analysis. Mater. Corros..

[18] Al-Obaid Y. (1995). The effect of shot peening on stress corrosion cracking behaviour of 2205-duplex stainless steel. Eng. Fract. Mech..

[19] Łabanowski J., Ossowska A. (2006). Influence of burnishing on stress corrosion cracking susceptibility of duplex steel. J. Achiev. Mater. Manuf. Eng..

[20] Wickström L., Mingard K., Hinds G., Turnbull A. (2016). Microcrack clustering in stress corrosion cracking of 22Cr and 25Cr duplex stainless steels. Corros. Sci..

[21] ASTM International (2006). Standard Practice for Evaluation Stress-Corrosion-Cracking Resistance of Metals and Alloys in a Boiling Magnesium Chloride Solution.

[22] Noyan I., Cohen J. (1987). Residual Stress Measurement by Diffraction and Interpretation.

[23] Lyon K., Marrow T., Lyon S. (2015). Influence of milling on the development of stress corrosion cracks in austenitic stainless steel. J. Mater. Process. Technol..

[24] Lu J., Luo K., Yang D., Cheng X., Hu J., Dai F., Qi H., Zhang L., Zhong J., Wang Q., Zhang Y. (2012). Effects of laser peening on stress corrosion cracking (SCC) of ANSI 304 austenitic stainless steel. Corros. Sci..

[25] Moverare J., Odén M. (2003). Influence of elastic and plastic anisotropy on the flow behavior in a duplex stainless steel. Metall. Mater. Trans. A.

[26] Peng R., Wang Y.D., Chai G.C., Jia N., Johansson S., Wang G. (2006). On the development of grain-orientation-dependent and inter-phase stresses in a super duplex stainless steel under uniaxial loading. Mater. Sci. Forum.

[27] Liu B., Feng X., Zhang S.-M. (2009). The effective Young’s modulus of composites beyond the Voigt estimation due to the Poisson effect. Compos. Sci. Technol..

[28] Peng R.L., Gibmeier J., Chai G., Johansson S. Load partitioning in a duplex strainless steel with surface strength gradient and residual stresses. Proceedings of the 57th Annual Conference on Applications of X-ray Analysis and the 8th International Conference on Residual Stresses, Advances in X-Ray Analysis.

[29] NACE International (2016). Four-Point Bend Testing of Materials for Oil and Gas Applications.

[30] International Organization for Standardization (2015). Corrosion of Metals and Alloys—Methodology for Determining the Resistance of Metals to Stress Corrosion Cracking Using the Four-Point Bend Method.

[31] Turnbull A., Mingard K., Lord J., Roebuck B., Tice D., Mottershead K., Fairweather N., Bradbury A. (2011). Sensitivity of stress corrosion cracking of stainless steel to surface machining and grinding procedure. Corros. Sci..

